# Seasonal variation of mortality, detectability, and body condition in a population of the adder (*Vipera berus*)

**DOI:** 10.1002/ece3.5166

**Published:** 2019-04-15

**Authors:** Dirk Bauwens, Katja Claus

**Affiliations:** ^1^ Department of Biology, Laboratory of Functional Morphology University of Antwerp Wilrijk Belgium

**Keywords:** capture–recapture, predation, seasonal variation, survival, vulnerability

## Abstract

We analyzed seasonal variation in mortality rates in adult males and females of the European adder (*Vipera berus*), using data collected during a 13‐year capture–recapture study (2005–2017) in a large population. We concurrently obtained quantitative information on the seasonal variation in the detectability and body condition of adders. Our results show strong seasonality in body condition, encounter, and capture rates of adult adders, and these patterns differ markedly between sexes and between breeding and nonbreeding females. Seasonal variation in mortality rates was however virtually nonexistent in males and moderately low in both breeding and nonbreeding females. In addition, we found no evidence for among‐year differences in the seasonal mortality schedules of males and females. During periods of intensive basking, both males and pregnant females are highly visible for humans, but are not subject to strong natural mortality. This low susceptibility to predation is presumably induced by various factors, including the limitation of overt exposure to short periods of time and specific microhabitats, the dorsal coloration pattern that provides cryptic protection and possibly also an aposematic warning signal, and presumed seasonal differences in the foraging behavior and food requirements of natural predators. Our data provide some evidence that female adders, but not males, are relatively vulnerable to predation during the seasonal migrations between the hibernation and feeding habitats. Mortality in the females was not much elevated during their breeding years, but was notably highest in the spring of the ensuing nonbreeding year. After giving birth, reproductive females are extremely emaciated and have a weakened general condition. They then run the risk of dying from starvation either before, during, or after hibernation. The higher mortality after giving birth, that is sustained over a period of ca. 9 months, should be considered as an indirect and delayed survival cost of reproduction.

## 1 INTRODUCTION

Survival of individual organisms is a fundamental component of population dynamics. Thus, the quantification of survival rates and identification of the processes and factors that affect survival are central to understanding fluctuations in population size. They also provide crucial information for adjusting management and conservation actions of (endangered) populations (Beissinger & Westphal, 1998; Boyce, 1992; Caughley, 1977). Constructing mortality schedules is often challenging, because survival rates may vary through time, space, and among age and sex classes within a population. In addition, multiple mortality agents (e.g., predation, parasitism, disease, starvation) contribute to observed rates of survival. Partitioning of these sources of mortality is notoriously difficult because actual mortality events are rarely observed in natural populations, such that one has to rely on indirect evidence to estimate the magnitude of putative mortality causes.

Snakes pose a particular problem for population studies as many species have complex life cycles and secretive habits, making them difficult to detect and observe in the field (Durso & Seigel, 2015; Steen, 2010). This complicates field studies by mark–recapture procedures, a standard method for obtaining population parameters. Nevertheless, several long‐term efforts have succeeded in obtaining robust empirical data on survival rates and other population parameters (Baron, Galliard, Tully, & Ferrière, 2010; Bauwens & Claus, 2018; Bonnet, Lourdais, Shine, & Naulleau, 2002; Brown, Kéry, & Hines, 2007; Lourdais, Bonnet, Shine, et al., 2002; Madsen & Shine, 1994; Weatherhead, Blouin‐Demers, & Sperry, 2012). In addition, temperate zone snakes exhibit pronounced seasonal patterns of activity rhythms and sex‐specific behaviors. Several aspects associated with distinct reproductive and other activities have presumed or demonstrated effects on mortality risks in various species. For instance, the increased mobility associated with mate searching by males is expected to put them at higher risk of predation and could result in elevated mortality during the mating season (Madsen & Shine, 1993; Sperry & Weatherhead, 2009). Seasonal increases of overt basking behavior are observed in males and/or females of various species and could augment their vulnerability to predators (Bonnet et al., 2002; Madsen & Shine, 1992; Sperry & Weatherhead, 2009). Females of live‐bearing viperids reproduce with a less‐than‐annual frequency; they invest heavily in the clutch, reduce, or even cease feeding during the 2–3 months pregnancy period (Bauwens & Claus, 2019a; Bonnet, Naulleau, Shine, & Lourdais, 2001; Lourdais, Bonnet, & Doughty, 2002; Madsen & Shine, 1992; Prestt, 1971) and are very emaciated after parturition (Bonnet et al., 2002; Madsen & Shine, 1993). They face high mortality risks, either through starvation, or because they are taken by predators while foraging (Bonnet et al., 2002; Madsen & Shine, 1993). Thus, the marked seasonality of reproductive and other behaviors provides an opportunity to infer the presumed effect of specific mortality agents on survival abilities of certain population segments.

We here report on the extent and putative causes of seasonal variation in mortality rates in adult males and females of the European adder (*Vipera berus*). We use data collected during a capture–recapture study conducted over a 13‐year period (2005–2017) in a large population of adders in northern Belgium. This citizen science project succeeded in obtaining demographic data for a large sample of individually marked adders (Bauwens & Claus, 2018). Our present aims are threefold.

First, to quantify seasonal differences in survival rates in adult adders. Because there are obvious sex‐bound differences in activity periods and behaviors, we a priori decided to separately analyze data for males and for females in their breeding and nonbreeding years. We use likelihood‐based analytical methods to explicitly account for the variation in capture rates, a necessary procedure to obtain robust estimates of survival probabilities (Cooch & White, 2015; Lebreton, Burnham, Clobert, & Anderson, 1992; Mazerolle, 2015).

Second, to provide quantitative information on seasonal variation in the detectability and body condition of adders, two characteristics that integrate a suite of underlying behavioral and ecophysiological traits. Estimates of encounter and capture rates quantify the detectability (or “visibility”) of adders to human snake catchers. These metrics incorporate seasonal variation in activity rhythms, mobility, and thermoregulatory behaviors that are presumably induced by hormonal and physiological changes associated with reproduction. The body condition index (mass relative to body length) is sensitive to the amount of fat stores and reflects the balance between the energy investments in reproductive activities and the energy gains by food intake during the foregoing months (Bonnet et al., 2002; Forsman & Lindell, 1997; Nilson, 1981).

Third, to examine to what extent seasonal differences in survival rates were congruent with variation in detectability, body condition, and other aspects of the ecophysiology and natural history of this species. This might enable us to identify putative mortality agents and processes.

## 2 MATERIALS AND METHODS

### 2.1 Study species and phenology

The European adder (*Vipera berus*) is a small, stout‐bodied venomous snake that has a huge distribution area covering large parts of Europe and Asia. They typically occur in small, often imperiled populations (10–100 adult individuals; Madsen et al., 2000; Madsen, Stille, & Shine, 1996; Phelps, 2004; Ursenbacher & Monney, 2003). By contrast, in our study area adders are very abundant; total population size is estimated to be in the order of several thousand snakes.

Most adders have a characteristic dark, zig‐zag vertebral band on a greyish (males) or brownish (females) background. The contrast between the zig‐zag markings and the background is most pronounced in the males, especially after they slough their skin during spring; females are generally duller in overall color contrast and may also display a more straight‐edged vertebral band (Prestt, 1971; Shine & Madsen, 1994; Lindell & Forsman, 1996; own observations).

The annual cycle of the adder in our study area coincides generally with that observed in other regions (Andrén, 1985; Madsen & Shine, 1993; Madsen, Shine, Loman, & Hakansson, 1993; Nilson, 1980; Phelps, 2004; Prestt, 1971; Völkl & Thiesmeier, 2002). Briefly, male adders emerge from hibernation at the end of February or beginning of March. They stay close to their winter dens in the so‐called hibernation area (Prestt, 1971) for about one month, spend much time basking to boost the production of spermatozoa and do not feed during this period. This period ends when they shed their skin around mid‐April and begin to move around in search of females, and engage in courtship and ritualized male–male combat. The mating season runs until mid‐May, when the adult males set off to the feeding or so‐called “summer” grounds (Prestt, 1971). Females and immatures emerge from hibernation around mid‐March; soon hereafter immature snakes and nonreproductive females move out to the feeding areas. Breeding females spend the summer in the hibernation areas, carefully thermoregulating to enhance the development of the embryos. The fully developed young are born during the second half of August or the beginning of September. Snakes return to the hibernation habitats during September–October and enter hibernation soon thereafter.

At our study site, newborn snakes measure 13–16 cm snout‐vent length (SVL); adult males and females reach maximal SVLs of 55 and 60 cm, respectively. Females produce their first litter upon attaining 38–40 cm SVL at the age of 3 or 4 years (i.e., in their fourth or fifth activity season). They are typical capital breeders that initiate reproduction upon surpassing a threshold level of energy stores, which are invested into the offspring. The depleted energy reserves are restored during the following year(s) (Bauwens & Claus, 2019b). As reproduction takes place on a less‐than‐annual basis, adult females with breeding and nonbreeding status are simultaneously present in the population. Litter size varies between 4 and 12 young and increases with female size.

### 2.2 Study area and data collection

Data were collected during a long‐term citizen science population study (2000 – 2017) of adders in the “Groot Schietveld” (ca. 1,570 ha; N 51°20–22′–E4°32–37′, province of Antwerp, Belgium). The area is used (since 1893) as a military exercise zone, and access is restricted to authorized persons and only when there are no military activities (mainly during nonworking days and hours). This lowland area (altitude ranges 18–25 m above sea level) is covered by a mosaic of heathlands, moors, fens, and woodlands. It is an isolated remnant of the vast extension of heathlands that once covered northern Belgium and is now entirely surrounded by agricultural land and residential areas that are totally unfavorable for adders. The nearest adder sites are located at 18 km SSE (a small imperiled population), 110 km E, 115 km NE, and 130 km S.

Adders are found over the entire military domain, but we concentrated our searches to 11 study plots (1–8 ha each; total search area: 46.5 ha) that were chosen for high snake encounter rates, ease of access, no direct impact of military operations, and their dispersed location over the area (mutual straight‐line distances between the geographic centroids of the plots range between 280 m and 6,800 m, mean = 2,685 m, *SE* = 197 m). Adders very occasionally moved between nearby study sites (Bauwens & Claus, in prep.), but the (meta)population in its entirety is effectively isolated. Immigration from other areas is nonexistent, and permanent emigration out of our study area is likely to result in mortality, given that the surrounding habitats are unfavorable for adders. Search areas are typical hibernation habitats and covered by a dense (percent groundcover typically >95%) vegetation dominated by dwarf shrubs of common heather (*Calluna vulgaris*), cross‐leaved heath (*Erica tetralix*) and bog‐myrtle (*Myrica gale*), tussocks of purple moor grass (*Molinia caerulea*), patches of mosses, and some localized thin groups of birch (*Betula pubescens*) and pine (*Pinus sylvestris*). From 2011 onwards, we also searched in four feeding habitats, mainly consisting of rough abandoned farmland, that were located 290–460 m distant from the corresponding hibernation grounds.

We visited each site several times per year, during favorable weather and throughout the adders' active season (late February to late October). For every visit to a sample locality, we recorded date, start and end time, and number of trained field assistants participating. This enabled calculation of the number of person‐hours per day (or any other period), our index of capture effort (Table 1).

**Table 1:** Number of unmarked adders released and number of them that were recaptured (in parenthesis) per seasonal period per year for adult males and females

	2005	2006	2007	2008	2009	2010	2011	2012	2013	2014	2015	2016	2017
Males													
Early spring	24 (9)	23 (13)	20 (7)	59 (17)	31 (14)	30 (12)	39 (17)	65 (42)	51 (35)	75 (44)	71 (40)	64 (38)	86 (23)
Late spring	5 (0)	24 (10)	2 (1)	6 (3)	3 (0)	2 (1)	4 (3)	12 (4)	22 (13)	3 (0)	8 (2)	24 (15)	23 (5)
Summer	13 (4)	2 (0)	13 (8)	6 (2)	6 (3)	7 (1)	13 (9)	21 (15)	4 (1)	17 (11)	7 (3)	24 (10)	18 (1)
Autumn–winter	4 (3)	8 (5)	29 (14)	12 (7)	8 (4)	8 (5)	19 (13)	17 (14)	21 (15)	32 (22)	19 (10)	25 (10)	17 (0)
Yearly total	46 (16)	57 (28)	64 (30)	83 (29)	48 (21)	47 (19)	75 (42)	115 (75)	98 (64)	127 (77)	105 (55)	137 (73)	144 (29)
Females													
Spring	17 (8)	2 (1)	7 (5)	12 (3)	12 (4)	7 (3)	6 (3)	28 (15)	26 (20)	29 (18)	30 (25)	18 (12)	29 (18)
Summer	13 (6)	13 (5)	33 (14)	16 (3)	10 (2)	23 (8)	39 (16)	47 (19)	44 (26)	37 (20)	34 (16)	56 (18)	68 (1)
Autumn–winter	6 (0)	8 (4)	20 (6)	10 (3)	2 (0)	8 (3)	11 (6)	16 (10)	20 (11)	25 (11)	15 (3)	30 (10)	5 (0)
Yearly total	36 (14)	23 (10)	60 (25)	38 (9)	24 (6)	38 (14)	56 (25)	91 (44)	90 (57)	91 (49)	79 (44)	104 (40)	102 (19)
Effort (person‐hours)													
Early spring	24.3	25.9	28.7	36.5	34.8	22.0	48.5	132.7	87.7	178.3	133.8	135.8	125.2
Late spring	12.0	14.3	5.3	8.1	5.3	10.3	12.5	53.7	60.7	49.5	43.1	42.9	76.3
Summer	53.9	24.6	59.2	32.5	31.4	35.3	102.5	187.2	129.6	171.7	149.2	183.0	165.1
Autumn–winter	38.4	36.2	66.4	33.4	22.1	28.0	91.5	137.7	145.8	175.3	129.5	126.9	110.9
Yearly total	128.6	101.0	159.6	110.5	93.5	95.7	255.0	511.2	423.8	574.7	455.6	488.6	477.5

Notes

Data for 1,146 individual males summarize 1,691 seasonal encounters, derived from 2,638 captures; data for 832 individual females summarize 1,494 seasonal encounters, derived from 1,798 captures. The bottom rows give seasonal and yearly indices for capture effort (number of person‐hours spent in the field).

Snakes were located by sight while walking slowly and erratically through the terrain, captured by hand, and released immediately after handling. A digital photograph of the upper side of the head allows individual identification of adders, on the basis of the number, shape, and arrangement of the head scales (Bauwens, Claus, & Mergeay, 2018). At every encounter, we also recorded date, time, exact location (GPS coordinates), sex, SVL (to the nearest 5 mm), body mass (to the nearest 1 g), and indications of recent food intake (midbody swelling, excretion of feces). Gender was determined on the basis of color, color pattern, and body proportions, and in the immatures by the number of subcaudal scales. Recently shed skins found in the field were collected and, when head scales were well preserved, assigned to an individual snake.

Yearly reproductive status of individual adult females (reproductive/breeding vs. nonreproductive/nonbreeding) was assessed by palpation of the abdomen to detect oviductal eggs or developing embryos and/or by signs of postparturient body condition (i.e., the presence of flaccid abdomen and extensive skin folds). To avoid the mistaken assignment of nonbreeding in a given year, this status was assigned only to females that did not show signs of pregnancy during June–August or had no indications of recent parturition in September–October.

The analyses that we report here deal exclusively with adult snakes. Adders were considered as adults when they were in at least their third (males) or fourth (females) active year. Aging assessments of individual snakes were based on their previous capture history and/or on SVL‐criteria inferred from the growth trajectories of adders that were initially caught in their first or second activity season (detailed description in Bauwens & Claus, 2018).

### 2.3 Delimitation of seasonal periods

On the basis of the adders' phenology, we distinguished among three (females) or four (males) seasonal sampling periods. The spring period (1 March–15 May) coincides with male spermiogenesis, mating, and female vitellogenesis. For the analysis of the male data, this period was split into early spring (1 March–15 April), the main basking period (mean shedding date during 2000–2017 was 12 April), and late spring (16 April–15 May), the mating period. During summer (16 May–15 August) reproductive females are gravid and stay in the hibernation areas, while males and nonbreeding females reside in the feeding habitats. In autumn–winter (16 August–30 October–28 February) reproductive females give birth to the young and resume feeding, males and nonbreeding females return to their hibernation habitats, and all adders enter hibernation. Hibernating adders could not be monitored, such that autumn and winter periods were lumped.

### 2.4 Modeling of capture and survival probabilities

Our analyses are based on the data collected during an intensive capture–mark–recapture program that generated 4,436 encounters of identified snakes (Table 1). Because of obvious differences in activity periods and behaviors between males and females, they were a priori expected to differ in the temporal variation for all traits considered here. We therefore analyzed them separately.

We estimated seasonal and among‐year variation in capture and survival rates using Cormack–Jolly–Seber (CJS) and Multistate capture–recapture methods (Cooch & White, 2015; Lebreton et al., 1992; Powell & Gale, 2015). Previous analyses of among‐year variation in age‐dependent survival rates in our study population (Bauwens & Claus, 2018) showed that estimates were quite imprecise and unstable during the initial years of the study (i.e., 2000–2004), concomitant with lower sampling efforts. To avoid obtaining spurious estimates on a more fine‐grained, that is, seasonal timescale, we restricted analyses to the data collected in the years 2005–2017.

During initial data handling, we aggregated the daily capture data of individual snakes into seasonal sampling periods. In male adders, the encounter history file for each individual snake included a single entry (1/0; captured/ not captured) per combination of season and year. In female adders, we included their annually fixed breeding status into the encounter history files, such that they contained for each individual female one entry (B/N/0; Breeding/ Nonbreeding/ not captured) per combination of season and year. Data aggregation in seasonal periods implicitly and erroneously assumes that sampling was instantaneous within each period, while it was actually spread over periods of up to three months. However, simulation studies showed that this does not have a strong effect on parameter estimates obtained by the CJS method (Hargrove & Borland, 1994). We tested the main assumptions of the capture–recapture methods by examining the goodness of fit of the data to the general time‐dependent models, using the programs RELEASE (Burnham, Anderson, White, Brownie, & Pollock, 1987) and U‐CARE 2.3.4 (Choquet, Lebreton, Gimenez, Reboulet, & Pradel, 2009).

We fitted the general time‐dependent model and various constrained versions of it and used an information theoretic approach to rank models according to the sample‐size‐adjusted Akaike's Information Criterion (AIC_c_; Burnham & Anderson, 2002; Anderson, 2008). For male adders, the general time‐dependent model included the effects of season and year on capture and survival probabilities. We did not expect the seasonal patterns to differ among years, so we included only additive effects. On the basis of the females' behavior and biology, we a priori hypothesized that seasonal patterns of capture and survival rates would differ between breeding and nonbreeding years. Hence, our initial model for females included the factors breeding state, season and year, an interaction effect between season and breeding state, and an additive effect of year.

To find the most parsimonious representation of the data, we used a step‐wise procedure (Burnham & Anderson, 2002; Lebreton et al., 1992). We started model selection by searching the most parsimonious structure for capture probability, while keeping maximal dimensionality for survival probability. For the females, we then modeled the transition probabilities between reproductive states, taking into account that an individual female can switch reproductive status between successive years, but never during the course of a given calendar year. Therefore, the transition probabilities between reproductive conditions were fixed to zero for the spring and summer periods. In a final step, the resulting parsimonious structures for capture and transition probabilities were kept to model survival rate in relation to season, year, and, in females, breeding status. Because the time intervals between consecutive seasonal periods were unequal, we included their duration (in months) in our model specifications, following the procedure outlined in (Cooch & White, 2015). This generated monthly survival estimates that are directly comparable among seasonal periods.

Parameter estimates for capture, transition, and survival probabilities were obtained by model averaging, weighted by model probability, over the entire model set (Anderson, 2008; Burnham & Anderson, 2002).

All models were fitted using maximum likelihood methods implemented in program MARK (Cooch & White, 2015; White & Burnham, 1999) through the RMark interface package (Laake & Rexstad, 2015) in R v3.5.1 (R Core & Team, 2018). All modeling specifications follow procedures outlined in Cooch and White (2015) and Laake and Rexstad (2015).

### 2.5 Encounter rate

In addition to the estimates of capture probability derived from the capture–recapture data, we obtained information on the seasonal variation in the detectability (or “visibility”) of adders to human snake catchers by estimating encounter rate, that is, the number of captures per person‐hour in a given time period. The high occurrence of visits to the area throughout the adders' active season allowed us to estimate encounter rate per half‐month, providing a time‐dependent index that is more fine‐grained than the seasonal capture rate index.

### 2.6 Body condition index

To obtain an index of body condition, we first calculated the baseline relation between logMass and logSVL for male and female adders separately, excluding measurements taken after recent food intake or recent parturition (males: logMass = −2.441 + 2.558 logSVL, *n* = 2,950, *R* ^2^ = 0.968; females: logMass = −2.678 + 2.732 logSVL, *n* = 2,297, *R* ^2^ = 0.968). Next, we estimated body condition at each capture occasion as the difference between the observed mass and the mass predicted by the baseline relation, a procedure that is equivalent to the calculation of regression residuals (Schulte‐Hostedde, Zinner, Millar, & Hickling, 2005). All analyses of body condition exclude records taken while signs of recent feeding were clearly visible.

## 3 RESULTS

### 3.1 Modeling of capture and survival probabilities

**Figure 1:**
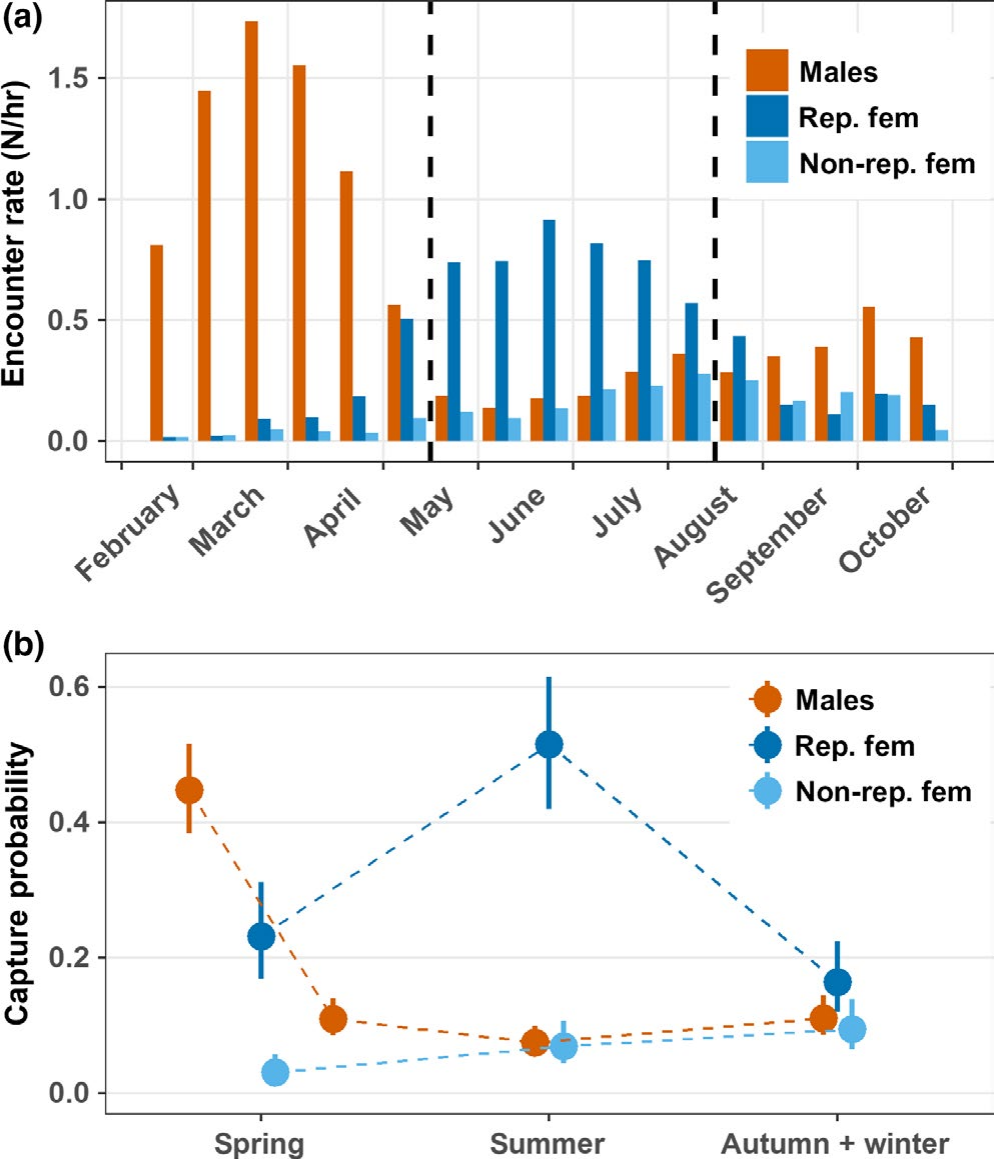
Seasonal patterns of detectability in male and in reproductive (“rep. fem.”) and nonreproductive (“non‐rep. fem.”) female adders. (a) Encounter rate (number of adders encountered/field hour) per half‐month, averaged over the years 2005–2017. The vertical dashed lines denote separation among the three main seasonal periods. (b) Capture rates per season as obtained by analyses of the capture–recapture data and calculated by weighted averaging over the entire model set listed in Tables 2 and 3, part (a). In the adult males, the spring season was divided into an “early” and “late” period (see text). Shown are the mean (dots) and 95% confidence limits (vertical lines) for the years 2005–2017

**Figure 2:**
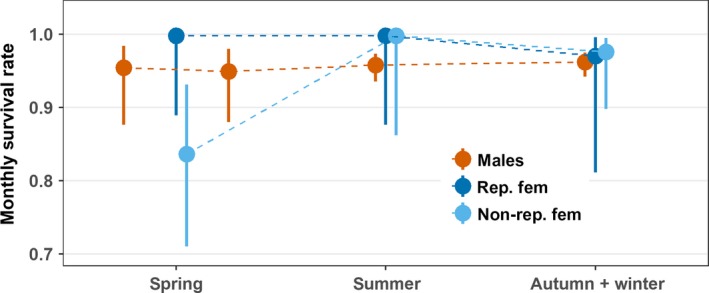
Estimates of the mean monthly survival probabilities during distinct seasonal periods in males, reproductive (“rep. fem.”), and nonreproductive (“non‐rep. fem.”) females. Estimates were obtained by weighted averaging over the model set listed in Table 2, part (b) (males) and Table 3, part (c) (females). The vertical lines show the 95% confidence intervals

The encounter data for 1,146 individual male adders showed a good fit to the general time‐dependent CJS model (program RELEASE: Test2: Chi^2^ = 98.5, *df* = 110, *p* = 0.78; Test3: Chi^2^ = 57.7, *df* = 85, *p* = 0.99; cumulative test: Chi^2^ = 156.3, *df* = 195, *p* = 0.98). The model of capture probabilities that was retained as the most parsimonious one, included the effects of season and year (Table 2, model 5). Capture rates in the males varied dramatically among seasons; they were notably higher during early spring than in the other periods (Figure 1b). During all seasons, capture probabilities were higher in the last years of the study (2011–2017) than in the preceding years (2005–2010), reflecting a year‐round increase in sampling effort (Table 1). The most parsimonious models for survival probabilities either assumed constant survival among seasons and years (Table 2, model 6), or variation among seasons, but not among years (Table 2, model 7). Accordingly, monthly survival rates were highly similar in the distinct seasons, with overlapping confidence intervals (Figure 2).

**Table 2:** Results of modeling of capture (*p*) and survival probabilities (Phi) in adult male adders obtained by CJS methods

Number	p	Phi	*K*	AIC_c_	ΔAIC_c_	Weight
(a) Modeling of capture probabilities						
**5**	**Season + year**	**Season + year**	**32**	**6,609.0**	**0.0**	**1.000**
3	Season	Season + year	20	6,709.5	100.6	0.000
4	Year	Season + year	29	7,148.8	539.9	0.000
2	Effort	Season + year	18	7,195.6	586.7	0.000
1	Constant	Season + year	17	7,252.1	643.2	0.000
(b) Modeling of survival probabilities						
**6**	**Season + year**	**Constant**	**17**	**6,597.6**	**0.0**	**0.716**
**7**	**Season + year**	**Season**	**20**	**6,599.5**	**1.9**	**0.273**
8	Season + year	Year	29	6,606.5	9.0	0.008
5	Season + year	Season + year	32	6,609.0	11.5	0.002

Notes

Based on the capture–recapture histories of 1,146 individual adders. Two successive steps of model selection were conducted (see text). At each step, the most informative models according to the AIC_c_ are indicated in bold when ΔAIC_c_ was <2.0. To obtain monthly survival probabilities that are directly comparable among seasonal periods, we specified unequal time intervals between consecutive seasons (i.e., 1.25, 2, 3.75, and 5 months). Shown are the model name (number), model structure for capture and survival probabilities (the “+” sign denotes an additive effect between two variables), the number of estimated parameters (*K*), Akaike's information criterion corrected for small sample size (AIC_c_), the difference in AIC_c_ between each model and the most parsimonious model (ΔAIC_c_), and the “Akaike weights” (weight) that assess the support that a given model has from the data, compared with the other models in the set.

We built recapture histories for 832 individual females and analyzed them with a multistate capture–recapture model. The encounter data provided an adequate fit to the general time‐dependent multistate model (program U‐care: test 3G: Chi^2^ = 92.6, *df* = 172, *p* = 0.99; test M: Chi^2^ = 101.6, *df* = 78, *p* = 0.04; cumulative test: Chi^2^ = 194.3, *df* = 250, *p* = 0.99). The model for capture rate that received most support of the data included the anticipated interaction effect between season and breeding status (Table 3, model 9). Capture rates of breeding females peaked during summer, whereas they were very low in the nonreproductive females during all seasons (Figure 1b). Transition probabilities differed between reproductive states (Table 3, model 9); mean annual estimates were higher for the transition from breeding to nonbreeding (Psi = 0.946, 95% CI = 0.906–0.970) than for the reverse shift (Psi = 0.585, 95% CI = 0.459–0.701). The most parsimonious model for survival probabilities included the interaction between breeding state and season, but no among‐year variation (Table 3, model 12). During reproductive years, monthly survival rates were maximal during the spring and summer periods and were slightly lower in autumn. In the nonbreeding years, monthly survival rates were lowest during the spring season (Figure 2). They were also most variable in spring, presumably due to the low capture rates during that season (Figure 1).

**Table 3:** Results of modeling of capture (*p*), transition between reproductive states (Psi), and survival probabilities (Phi) in adult female adders obtained by multistate mark–recapture methods

Number	*p*	Psi	Phi	*K*	AIC_c_	ΔAIC_c_	Weight
(a) Modeling of capture probabilities							
**9**	State * season + year	**State**	State * season + year	**38**	**4,115.1**	**0.0**	**1.000**
7	State * season	State	State * season + year	26	4,153.2	38.1	0.000
8	State + year	State	State * season + year	34	4,265.1	149.9	0.000
10	State * Effort	State	State * season + year	24	4,290.2	175.1	0.000
6	State	State	State * season + year	22	4,290.2	175.1	0.000
4	Year	State	State * season + year	33	4,450.7	335.6	0.000
3	Constant	State	State * season + year	21	4,516.6	401.5	0.000
5	Effort	State	State * season + year	22	5,363.9	1,248.7	0.000
2	Season + year	State	State * season + year	35	5,445.2	1,330.1	0.000
1	Season	State	State * season + year	23	5,654.6	1539.5	0.000
(b) Modeling of transition probabilities							
**9**	State * season + year	**State**	State * season + year	**38**	**4,115.1**	**0.0**	**1.000**
11	State * season + year	Constant	State * season + year	37	4,148.1	33.0	0.000
(c) Modeling of survival probabilities							
**12**	State * season + year	**State**	State * season	**26**	**4,104.9**	**0.0**	**0.916**
16	State * season + year	State	Season	23	4,111.8	6.8	0.030
13	State * season + year	State	Constant	21	4,111.8	6.9	0.030
15	State * season + year	State	State	22	4,113.2	8.2	0.015
9	State * season + year	State	State * season + year	38	4,115.1	10.8	0.005
17	State * season + year	State	Season + year	35	4,117.0	12.1	0.002
18	State * season + year	State	Year	33	4,118.4	13.4	0.001
14	State * season + year	State	State + year	34	4,120.4	15.4	0.000

Notes

Based on the capture–recapture histories of 832 individual adders. Three successive steps of model selection were conducted (see text). At each step, the most informative models according to the AIC_c_ are indicated in bold when ΔAIC_c_ was <2.0. To obtain monthly survival probabilities that are directly comparable among seasonal periods, we specified unequal time intervals between consecutive seasons (i.e., 2.5, 3, and 6.5 months).

Shown are the model name (number), model structure for capture, transition, and survival probabilities (the “*” sign denotes an interaction effect and the “+” sign an additive effect between two variables), the number of estimated parameters (*K*), Akaike's information criterion corrected for small sample size (AIC_c_), the difference in AIC_c_ between each model and the most parsimonious model (ΔAIC_c_), and the “Akaike weights” (weight) that assess the support that a given model has from the data, compared to the other models in the set.

### 3.2 Temporal patterns of encounter rate

In the adult males, the encounter rate was highest from half March to half April and then decreased to attain minimal levels during the second half of May (Figure 1a). It remained low throughout the months of June to August and increased somewhat near the end of the activity period. This pattern is very similar to the seasonal variation in capture rates (Figure 1b).

We note that the early spring encounters of males included the finds of 148 skin molts that, upon examination of the head scalation pattern, could be assigned to individual adders. In 83 cases (ca 5% of all spring encounters), these molts provided the only evidence of an individual's presence in that year.

The encounter rate of breeding females was low during March–April, when they were previtellogenic, peaked during the summer gestation period and dropped again in September–October, immediately after parturition (Figure 1a). The pregnant females' behavior during summer was analogous to that of the males during the early spring or basking period.

Adult females are extremely secretive and apparently absent throughout their nonreproductive years, as indicated by the very low encounter and capture rates (Figure 1).

### 3.3 Body condition index

**Figure 3:**
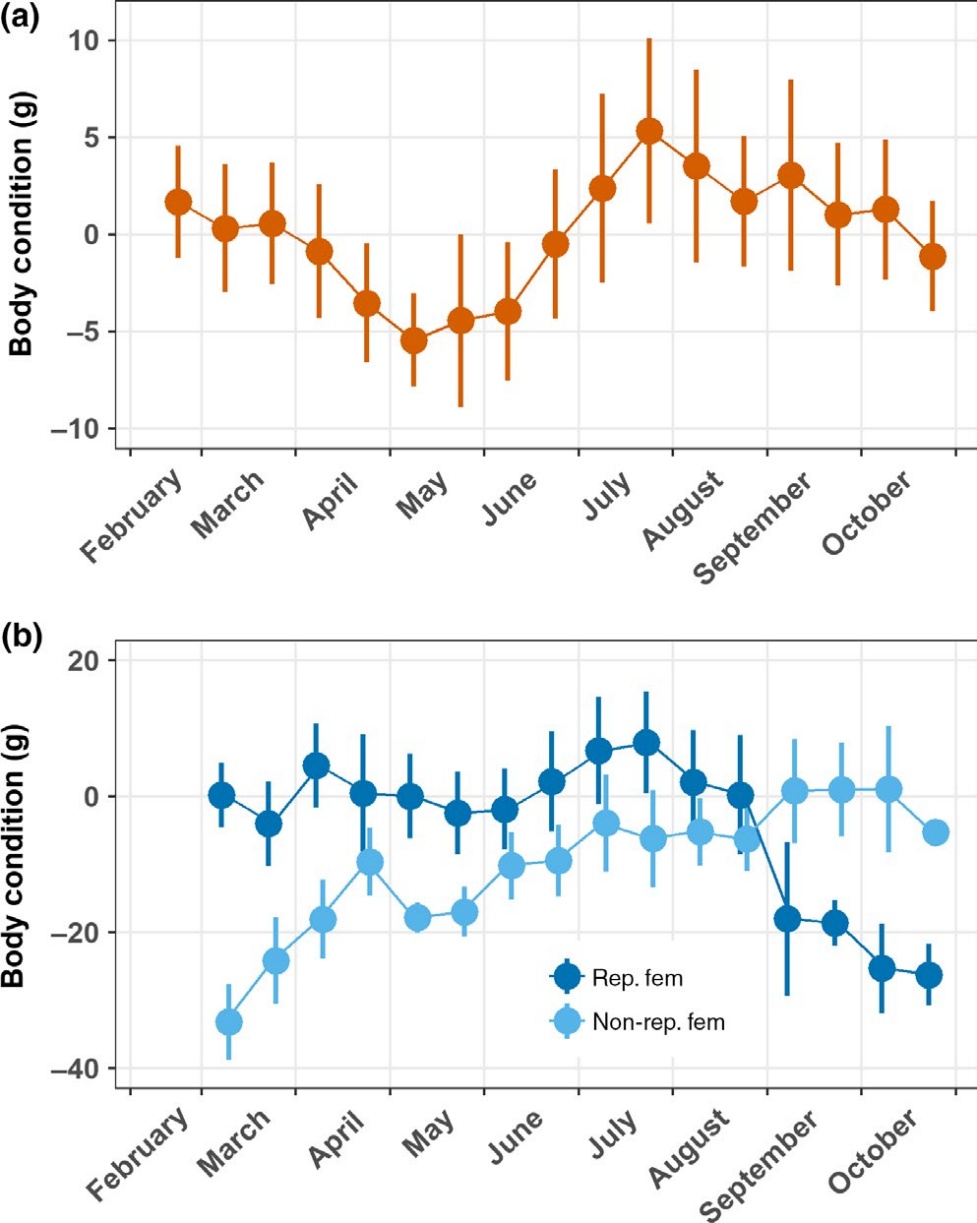
Seasonal patterns of body condition index in (a) adult male adders and (b) adult female adders during reproductive (“rep. fem.”) and nonreproductive (“non‐rep. fem.”) years. Shown are the median (dot) and interquartile range (vertical line) per half‐month for the years 2005–2017

We found obvious and well‐defined differences in the annual patterns of body condition between males and females, and between breeding and nonbreeding female adders (Figure 3).

Male adders emerge from hibernation with a “standard” BCI (i.e., equal to zero). This is maintained until the onset of April, when the median BCI drops to attain a minimal value in May (Figure 3a). During this interval males loose on average 5 g body mass, representing ca 10% of their initial mass. Subsequently, BCI steadily increases until the end of July, when males have gained on average 10 g over the period June–July. During August–October, BCI slowly decreases to attain a “standard” value before the onset of hibernation.

The seasonal pattern of the BCI differs dramatically between adult females in breeding and nonbreeding years (Figure 3b). Reproductive females emerge from hibernation with a “standard” BCI, and roughly maintain this score until the end of August, when it drops abruptly upon giving birth to their young. Median loss of body mass upon parturition is ca 25 g or a decrease of ca 30% of their body mass at emergence from hibernation. The low BCI is maintained over the hibernation period. The nonreproductive females, most of which have given birth the preceding year, initially have low BCI. It steadily increases over the next few months to attain maximal values in September, at the end of the activity period. Thus, after giving birth females require the entire activity period, or longer, to restore their BCI to “standard” values.

### 3.4 Predators and other mortality agents

Only on very rare occasions (*n* = 6, during 3,875 person‐hours of fieldwork), we observed that adders were attacked by predators, demonstrating that it is difficult to observe predation in the wild. Somewhat more frequent (*n* = 25) were the finds of partially eaten corpses and of scarred individuals. The most frequently observed reputed predators of adders in our study area are buzzards (*Buteo buteo*), European polecat (*Mustela putorius*), and red fox (*Vulpes vulpes*). These are nonspecialized predators that opportunistically take reptiles as a supplementary food source. Hence, predation on adders is presumably largely erratic in our study area. We consider buzzards as the main predators. This is supported by observations of flying buzzards holding an adder in their claws and by the shape of scars on the adders' bodies that were seemingly formed by a bird of prey's claws. We also found partially eaten adder remains (*n* = 2) that were attributed to killing by a buzzard. From 2015 onwards, there was an increase in the number of short‐toed snake eagles (*Circaetus gallicus*), a highly specialized snake hunter, that roost in the area during the summer months. Other observed causes of mortality are trampling by roe deer (*Capreolus capreolus*) and traffic kills on the few gravel roads that cross the study area. Wild boar (*Sus scrofa*), which have a significant negative impact on adder populations (Graitson, Barbraud, & Bonnet, 2018), are absent from our study area. Some postparturient females are extremely emaciated due to starvation during pregnancy; in three instances, we found their emaciated carcasses just before or after hibernation.

## 4 DISCUSSION

Our study of seasonal variation in survival rates in adult adders confirms and extends published findings, most notably those exposed for a small, threatened population in southern Sweden (Madsen & Shine, 1992,1993,1994). By contrast, our long‐term (13 years) study addressed a very large adder population, leading to an extensive database of the capture–recapture histories of large numbers of adult adders. Our efforts were not restricted to periods when adders are relatively easily observed (e.g., springtime for males, summer for breeding females), but span the complete annual active cycle. We concurrently collected detailed data on seasonal variation in the detectability and body condition of adders, and explore their congruence with patterns of mortality to identify putative mortality agents and processes.

Our results show strong seasonality in body condition, and in encounter and capture rates of adult adders. In addition, the observed patterns differ markedly between sexes and between breeding and nonbreeding females. The seasonal and among‐group variations are presumably induced by underlying physiological and behavioral processes and are supposed to affect susceptibility to predation and other mortality agents. On the other hand, seasonal variation in monthly mortality rates was virtually nonexistent in males and moderately low in both breeding and nonbreeding females. In addition, we found no evidence for among‐year differences in the seasonal mortality schedules (or lack thereof) of males and females. Thus, we were unable to detect striking temporal differences in mortality rates, even though our sample sizes are very large for a terrestrial snake. This may render it extremely difficult to identify the specific causes and correlates of mortality.

In the following sections, we first provide a comprehensive overview of available information on seasonality of activity, reproduction, body condition, and survival rates in adult males and females. Subsequently, we explore how distinct internal and external factors may affect the adders' susceptibility to predation and other mortality agents.

### 4.1 Seasonality in adult males

During the early spring or basking period, males accelerate the production of sperm (Nilson, 1980) by attempting to raise their body temperature above ambient levels. They stay close to their hibernation dens and are very sedentary at specific and characteristic microhabitats that are repeatedly used by the same and different individuals (Madsen & Shine, 1993; Prestt, 1971; Viitanen, 1967). Especially during the first days following emergence from hibernation, and afterward when thermal conditions constrain the range of achievable body temperatures (Herczeg et al., 2007), males bask overtly with flattened body in the sun or are simply lying out during overcast weather. Quite often several males are lying together, touching each other. They are usually rather unwary and can be approached quite readily. These behaviors make the male adders highly conspicuous to snake catchers, resulting in high encounter and capture rates during the early spring period.

Although both encounter and capture rates are at peak levels during the early spring basking period, this does not imply that all males present are actually captured. Averaged over the years, the capture rate during the early spring period was ca 0.45, indicating that less than half of the males present were actually encountered then. The adult males' abilities to evade sighting and capture is also illustrated by the finds of identified shed skins from individual adders that went otherwise unnoticed to us during the early spring period or even the entire active year. These snakes were thus present during at least several weeks at our study sites, completed spermiogenesis, and shed their skin, but avoided being seen by us.

During the first half of April, the adult males finish their spring shedding cycle, coinciding with the end of spermiogenesis (Nilson, 1980). This induces abrupt changes in their visual appearance and in their behavior. The freshly sloughed males exhibit bright and vivid coloration, accentuating the contrast between the blackish zig‐zag dorsal stripe and the greyish background color. They increase their daily moving distance by a sevenfold (Madsen et al., 1993), disperse over a larger area in search of receptive females, and engage in ritualized male‐male combats (Madsen & Shine, 1993; Prestt, 1971; Viitanen, 1967). This should make them more conspicuous to human observers. However, in our study area encounter rates decreased quickly and capture rates attained very low levels during the late spring or mating period. Thus, detectability of mate‐searching males was not especially high in our study site, contrasting observations made in other areas (Andrén, 1985; Madsen & Shine, 1993; Prestt, 1971; Völkl & Thiesmeier, 2002). The behaviors associated with mating, as well as the physiological costs of sperm production are energetically costly (Madsen & Shine, 1993; Olsson, Madsen, & Shine, 1997). In addition, male adders do not feed during the entire spring period (Prestt, 1971; Völkl & Thiesmeier, 2002). As a result, they lose on average ca 10% of their initial body mass.

From the second half of May onwards, males move to the feeding grounds, where they forage (Prestt, 1971), resulting in a gradual restoration of their body condition. They disperse over a large area and a wide variety of densely vegetated habitat types, and spend much time in deep cover and even below ground (Hand, 2018). This is reflected by the very low capture rate (ca. 0.1), indicating that we succeeded in capturing only one out of ten males that were present.

Most males return to the hibernation habitats during September. The low capture and encounter rates indicate that they disappear quickly in the wintering dens. Over the winter period there is, on average, no notable decrease of body condition.

The substantial variation in male behavior throughout the activity season is expected to induce temporal differences in risks of predation, and hence, mortality rates (Madsen, 2011; Madsen & Shine, 1993; Sperry & Weatherhead, 2009). By contrast, our results do not provide evidence for important seasonal variation in the monthly survival estimates.

### 4.2 Seasonality in adult females

Patterns of seasonal variation differed dramatically between females in their breeding and nonbreeding years for all characteristics studied.

In years that they will breed, females emerge from hibernation with a high BCI, compliant with the notion that a threshold level of energy reserves is necessary to start a reproductive cycle in our population (Bauwens & Claus, 2019b), similarly as in the closely related *V. ursinii* (Baron, Galliard, Ferrière, & Tully, 2013) and *V. aspis* (Naulleau & Bonnet, 1996). During the first active weeks and throughout the mating period, these females are highly sedentary (Andrén, 1985; Prestt, 1971; Viitanen, 1967) and behave inconspicuously, as indicated by the low encounter and capture rates. It is noteworthy that we only very rarely observed courtship and mating behaviors, which contrasts sharply with the numerous observations made by T. Madsen in his study population (Madsen, 2011; Madsen & Shine, 1992; Madsen, Shine, Loman, & Håkansson, 1992).

Following ovulation during the first half of May, the detectability of gravid females increases and stays high throughout the gestation period. Gravid adders stay in the hibernation areas, show extremely limited mobility (Madsen & Shine, 1992), and spend much time thermoregulating (Lourdais, Guillon, DeNardo, & Blouin‐Demers, 2013). Especially during suboptimal weather conditions, females were observed basking or lying out. By spending much time to overt thermoregulatory behaviors, gravid females increase their exposure to humans, as revealed by the high encounter and capture rates.

During pregnancy, most female adders cease feeding or take food only sporadically (Bauwens & Claus, 2019a; Bea, Braña, Baron, & Saint‐Girons, 1992; Nilson, 1981; Prestt, 1971). The females' mass and BCI decline over time due to mobilization of remaining fat stores and muscle proteins, to meet the demands of maintenance (Bonnet et al., 2001; Dupoué & Lourdais, 2014; Lourdais, Brischoux, Denardo, & Shine, 2004). This loss is countered by occasional feeding events and by the uptake of water that is allocated to the developing embryos (Lourdais, Lorioux, Dupoué, Wright, & DeNardo, 2015). The net result is that the average BCI of the breeding females remains roughly constant throughout the spring and summer periods. However, the physical burden presumably increases during pregnancy, due to the increasing mass of the embryos and the decrease in body mass by losses of fat stores and musculature (Lorioux, Lisse, & Lourdais, 2013).

The breeding females' BCI drops abruptly upon giving birth in the second half of August, when they lose on average ca 30% of their body mass, leading to substantial emaciation at parturition. The low BCI values are maintained over the winter period. The postparturient females are captured infrequently, because at least a fraction of them migrate temporarily to the feeding habitats.

The nonbreeding females, the majority of which have given birth in the preceding year, reside most of the active season in the feeding grounds. They disperse over a large area with densely vegetated habitats, have lowered thermal needs, and spend more time under cover (Lorioux et al., 2013). This results in a very low detectability, similar to that of the adult males in summer and that of the immature adders (Bauwens & Claus, 2018). Upon emerging from hibernation they have a low BCI. It increases gradually throughout the next months to attain maximal values in September, at the end of the active period. Thus, after a breeding year, females require a complete active period (or longer) to forage and rebuild their fat reserves.

Seasonal variations in mortality rates were detected during both the breeding and nonbreeding years. In reproductive females, mortality was estimated to be absent during the spring and summer periods, a result that concords with that of Madsen & Shine (1993). After giving birth and throughout the hibernation period, mortality was higher, although the increase was not as dramatic as observed in the population studied by Madsen and Shine (1993). The mortality rates were clearly highest during the spring period of the nonbreeding years, when females were recovering from the foregoing reproductive effort. Mortality was low in the summer of the nonbreeding years and increased somewhat in the ensuing autumn and winter period.

### 4.3 Low vulnerability to predation during basking

Reproductive processes induce increased demands of thermoregulation in adders. Spermiogenesis during early spring in males and embryogenesis during summer in adult females are associated with a higher and narrower range of selected temperatures, and with overt basking and lying out (Herczeg et al., 2007; Lourdais et al., 2013). The extensive thermoregulatory behaviors induce increased visibility toward human predators, as indicated by the high encounter and capture rates during these seasonal periods. It has often been suggested or assumed that this would also be indicative for an elevated susceptibility to other visual hunting predators (Andrén, 1985; Bonnet et al., 2002; Lorioux et al., 2013; Sperry & Weatherhead, 2009). However, our results do not indicate that increased basking leads to higher mortality rates in either males or females, confirming findings by Madsen and Shine (1993). Thus, although adders seem to be especially detectable for human snake catchers during periods of intensive basking, they are not subject to strong natural mortality. This has led to the suggestion that immobile adders are relatively invulnerable to and even ignored by natural predators (Andrén, 1985; Madsen, 2011; Madsen & Shine, 1993). An idea that is however not supported by studies showing considerable levels of avian predatory attacks on motionless plastic or clay models of various *Vipera* species (Andrén & Nilson, 1981; Niskanen & Mappes, 2005; Santos et al., 2014; Valkonen, Niskanen, Björklund, & Mappes, 2011; Wüster et al., 2004).

Various aspects of the adders' biology may explain the apparent low susceptibility to predation of basking adders. Our extensive field observations reveal that the highly eye‐catching and apparently prolonged overt basking and lying out is in fact limited to rather short periods of time. As soon as thermal conditions allow attainment of the preferred body temperatures, often after only 5–15 min of continuous sunshine, adders retreat under cover of a thin layer of vegetation. Sightings are far less evident then, except to (trained) human snake catchers that thoroughly inspect the adders' favorite microhabitats. Field observations also indicate that basking adders typically frequent specific spots that favor careful thermoregulation, but that also facilitate rapid retreat to a nearby (<1 m) refuge (see Lorioux et al., 2013 for similar observations in the aspic viper). In short, adders generally exhibit a very secretive lifestyle, exposing themselves only at specific sites and for short periods. While human snake catchers are able to quickly adjust the locations and timing of their searches, not all predators may exhibit such a functional response. This seems especially likely for nonspecialized predators that opportunistically catch adders while searching for other types of prey (Selås, 2001). This presumably contributes to the discrepancy between high catchability by human predators and low mortality rates.

The characteristic dorsal zig‐zag pattern of *Vipera* species is generally considered as a disruptive coloration that provides cryptic protection toward visual hunting predators (Andrén & Nilson, 1981; Santos et al., 2014; Shine & Madsen, 1994). Results of experiments using artificial replicas with viper‐like paintings have been interpreted as providing evidence that the zig‐zag coloration also has an aposematic function (Niskanen & Mappes, 2005; Valkonen et al., 2011; Wüster et al., 2004). That conclusion is however not supported by the absence of a difference in mortality rates between melanistic and normal colored male adders (T. Madsen, pers. comm.). In summary, the dorsal coloration mainly provides cryptic protection and thus prevents the individual from being seen by predators, but may also serve as a warning signal once the individual has been detected. This would deter predatory attacks especially from basking adders that overtly show their contrasting zig‐zag dorsal pattern.

Seasonal differences in the foraging behavior and food requirements of natural predators will also contribute to variation in the mortality rates of their prey. For instance, it has been documented that individual buzzards may specialize on adders, and they do so mainly to nourish their nestling young (Bijlsma, 2012; Naulleau, Verheyden, & Bonnet, 1997). Nestling buzzards have the highest food demands during the summer months, which may invoke a temporarily increase of the predation pressure on adders. In addition, buzzards are more likely to catch adders when they reside in their feeding grounds, where small mammals, the bulk food of both buzzards and adders, are most abundant (Selås, 2001). Thus, the foraging habits of predators may partly induce higher mortality at times and places where adders are least detectable to humans and thereby disrupt the putative concurrence between mortality risks and visibility to human snake catchers.

### 4.4 Mobility and mortality risks

Fitting with the idea that immobile adders are relatively invulnerable to predation Madsen (2011) and Madsen & Shine (1992, 1993) proposed that predators would mainly target moving adders. Hence, mortality rates are expected to be higher during periods with intensified adder mobility. Our results provide only partial support for such a relation.

Adult males exhibit higher levels of mobility during the mating period when they search actively for receptive females and engage in extended courtship and ritualized male–male combats. This is thought to affect the risk of mortality, either directly (e.g., through increased vulnerability to predation) or indirectly (e.g., through a decrease in body mass and the concomitant weakened body condition; Andrén, 1985; Madsen, 2011; Madsen & Shine, 1993). However, no increased monthly mortality rates of adult male adders were detected during the spring mating period.

Foraging behavior has also been suggested to invoke increased mobility in both males and females (Madsen & Shine, 1992,1993). However, although adders occasionally search actively for nest holes of small mammals and for ground‐breeding birds (Bea et al., 1992; Völkl & Thiesmeier, 2002), they are mainly sit‐and‐wait predators (Glaudas et al., 2019). They spend long periods lying in ambush under cover of vegetation and typically keep their body in a coiled position that is highly similar to the posture maintained by basking adders. Thus, we question whether foraging in itself is associated with elevated mobility and would result in a higher susceptibility to predation. However, adders forage mostly in their feeding habitats, where food is more abundant (Luiselli, Capula, Rugiero, & Anibaldi, 1994; Phelps, 2004; Prestt, 1971; Viitanen, 1967; Völkl & Thiesmeier, 2002). The hibernation and feeding grounds can be apart by distances of up to 1–2 km and often cross suboptimal habitats that do not provide adequate protective vegetation cover (Madsen & Ujvari, 2011; Prestt, 1971; Viitanen, 1967; Völkl & Thiesmeier, 2002). We therefore suggest that the seasonal migrations between the winter and summer habitats, rather than the foraging as such, might induce increased vulnerability to predation.

Adult males undertake these migrations at the end of the mating season and before the onset of the winter, but no elevated mortality rates were detected then. Breeding females migrate shortly after giving birth, when they experience a slight increase in mortality. The nonbreeding females move to the feeding grounds soon after emerging from hibernation and back to the hibernation areas during autumn. During both these periods, their monthly mortality rates are relatively elevated. Hence, these results support, at least in part (see below), the idea that female adders are relatively vulnerable to predation during their seasonal migrations.

### 4.5 Delayed mortality costs of breeding

Reproduction entails costs, including an increased risk of mortality (Shine, 1980; Williams, 1966). Increased mortality during reproductive years has been documented in the adder (Madsen & Shine, 1993) and the aspic viper (Bonnet et al., 2002). By contrast, annual mortality rates were not affected by the females' breeding status in the meadow viper (Baron et al., 2013) and in our adder population (Bauwens & Claus, 2019b). Also, distinct reproductive activities are supposed to entail different degrees of mortality risks (Bonnet et al., 2002; Madsen & Shine, 1993; Sperry & Weatherhead, 2009; Weatherhead et al., 2012). Although our data indicate temporal variation in mortality rates, the observed pattern does not fully comply with general expectations.

Pregnancy in viviparous squamates is associated with physiological and behavioral modifications that are likely to affect mortality risks. By devoting more time to thermoregulation (Andrén, 1985; Lorioux et al., 2013; Lourdais et al., 2013) and by the physical burden of the weight of the litter (Lorioux et al., 2013; Nilson, 1981), pregnant females are presumably exposed to higher predation risks (Bonnet et al., 2002). This idea is however not supported by our results and those of Madsen & Shine (1993), as mortality of breeding females was virtually absent during spring and summer (i.e., during vitellogenesis and pregnancy). Females thus seem to adopt behavioral adjustments that reduce their susceptibility to predation during pregnancy.

Nevertheless, mortality during breeding years increased somewhat after giving birth and was highest in the spring of their ensuing nonbreeding year. As we suggested, this can be partly attributed to the increased susceptibility to predation during migrations to the feeding grounds.

Probably more important is that postparturient females are extremely emaciated and have a weakened general condition, due to the high investment in vitellogenesis and reduced feeding during pregnancy. Unless they are able to feed, they run the risk of dying from starvation either before, during or after hibernation (Madsen & Shine, 1992,1993). The accidental finds of carcasses of emaciated females support this suggestion. The elevated mortality after giving birth, that is sustained over a period of ca. 9 months, should thus be considered as an indirect and delayed survival cost induced by pregnancy (Bonnet, Naulleau, Shine, & Lourdais, 1999; Madsen & Shine, 1992).

As already stated by Madsen & Shine (1992), the behavior of gravid female adders seems to be directed toward reducing the probability of immediate mortality, at the cost of increasing the mortality rate after giving birth. This strategy increases the probability of success of the current reproductive bout, but lessens the chances of surviving to a next breeding occasion. Accordingly, most female adders (ca. 70%) that attained sexual maturity reproduced only once in their lifetime (Bauwens & Claus, 2019b; Madsen & Shine, 1992).

## CONFLICT OF INTEREST

None declared.

## AUTHOR CONTRIBUTIONS

K.C. designed and initiated the study and supervised data collection. K.C. and D.B. collected the data. D.B. analyzed the data. D.B. and K.C. wrote the paper.

## Data Availability

Capture‐recapture histories: https://doi.org/10.5281/zenodo.2559514.
